# Dapagliflozin in peritoneal dialysis patients: a pilot study evaluating peritoneal membrane function

**DOI:** 10.1186/s12882-023-03429-2

**Published:** 2024-01-26

**Authors:** Zakaria Hamdan, Yusri Abdel-Hafez, Ahmad Enaya, Alaa Sarsour, Lubna Kharraz, Zaher Nazzal

**Affiliations:** 1 Internal Medicine Department, An-Najah National University Hospital, Box 7, Nablus, 707 Palestine; 2 Palestinian Ministry of Health, Nablus, Palestine; 3 Kidney and Dialysis Section, An-Najah National University Hospital, Nablus, Palestine; 4 Pathology and Medical Laboratory Sciences, An-Najah National University, Nablus, Palestine; 5 Department of Medicine, Faculty of Medicine and Health Sciences, An-Najah National University, Box 7, Nablus, 707 Palestine

**Keywords:** Peritoneal dialysis, Dapagliflozin, Ultra-filtration failure, SGLT-2 inhibitors

## Abstract

**Background:**

Patients taking SGLT-2 inhibitors may experience delayed peritoneal fibrosis, better ultrafiltration of water and toxins, and higher survival rates. We aimed to evaluate the possible effects of Dapagliflozin in changing the peritoneal solute transfer rate, reducing peritoneal glucose absorption, and, hence, increasing ultrafiltration.

**Methodology:**

A pilot pre-post interventional study was used to evaluate 20 patients on continuous ambulatory peritoneal dialysis (CAPD) enrolled in a one-month self-controlled study [Trial#: NCT04923295]. Inclusion criteria included being over 18, and having a Peritoneal Dialysis (PD) vintage of at least six months. All participants were classified as having high or average high transport status based on their Peritoneal Equilibrium Test with a D0/D4 > 0.39. and using at least two exchanges with 2.35% dextrose over the previous three months before enrollment.

**Results:**

Following the treatment, 13 patients had an increase in median D4/D0 from 0.26 [0.17–0.38] to 0.31 [0.23–0.40], while seven patients had a decline from 0.28 [0.17–0.38] to 0.23 [0.14–0.33]. Additionally, nine patients had a decrease in median D/P from 0.88 [0.67–0.92] to 0.81 [0.54–0.85], while 11 patients had an increase from 0.70 [0.6–0.83] to 0.76 [0.63–0.91].

**Conclusion:**

According to the findings of this study, Dapagliflozin usage in peritoneal dialysis patients did not result in a reduction in glucose absorption across the peritoneal membrane. Additionally, Dapagliflozin was also associated with a small increase in sodium dip, a decrease in peritoneal VEGF, and a decrease in systemic IL-6 levels all of which were not statistically significant. Further large-scale studies are required to corroborate these conclusions.

## Key messages

- Dapagliflozin in this Peritoneal dialysis patients did not show a statistically significant reduction in glucose absorption across the peritoneal membrane.
- A small increase in the one-hour Sodium dip was noted after one month of Dapagliflozin administration in Peritoneal dialysis patients, along with a reduction in systemic IL-6 and intraperitoneal VEGF
- The long-term effects on the longevity of the peritoneal membrane in patients on peritoneal dialysis warrant further investigations.

## Background

Peritoneal dialysis (PD), a common option in renal replacement therapy for individuals with End-Stage Renal Disease (ESRD), uses the peritoneum as a dialysis membrane [1] and relies on an osmotic gradient between blood and dialysate to shift salt and water from the vasculature to the dialysate, to be discarded [2]. The standard osmotic agent used in the peritoneal dialysate is glucose, which is usually absorbed by the patient in varying degrees depending on the characteristics and transporter status of the peritoneal membrane [3].

Patients with high or rapid peritoneal transporters usually have adequate peritoneal clearance on a standard Continuous Ambulatory Peritoneal Dialysis (CAPD) regimen. However, they frequently encounter difficulty with ultrafiltration due to the excessive reabsorption of glucose, which diminishes the osmotic gradient necessary for UF to occur [4]. Ultrafiltration Failure (UF) is defined as failure to achieve at least 400 ml of net ultrafiltration during a 4-h dwell using 4.25% dextrose [5]. Failure to provide adequate levels of either or both of these parameters accounts for approximately 18% of overall technique failure and eventual transfer to hemodialysis (HD) [6].

Little is known regarding the effects of sodium-glucose transport protein 2 (SGLT-2) inhibitors on patients undergoing regular peritoneal dialysis. However, very recently, a wild-type mice model has been used and treated with glucose-rich dialysate solution through a peritoneal catheter with and without Dapagliflozin to analyze structural and functional changes in the peritoneal membrane. They concluded that peritoneal health has improved with Dapagliflozin in glucose-rich peritoneal dialysis without developing a high-glucose transporter status [7]. Moreover, the use of oral SGLT-2 inhibitors has also resulted in reduced glucose uptake and, thus, increased ultrafiltration through murine peritoneum [8]. That study also proved that SGLT-2 receptors are expressed in the human peritoneum and Human Peritoneal Mesothelial Cells (HPMC) and that glucose consumption and uptake by HPMC in conditions with high glucose concentrations have decreased with SGLT-2 [8]. Based on the previous findings, the authors suggested that using SGLT-2 inhibitors for patients undergoing peritoneal dialysis might result in delayed peritoneal fibrosis, better ultrafiltration of water and toxins, and, eventually, a better survival rate [8].

At the time of the enrolment, there were no studies to our knowledge that included patients on peritoneal dialysis on SGLT-2 inhibitors. Considering the importance of ultrafiltration failure in peritoneal dialysis, we aimed to evaluate the possible effects of Dapagliflozin in changing the peritoneal solute transfer rate, reducing peritoneal glucose absorption, and, hence, increasing ultrafiltration. Additionally, we were interested in evaluating the possible anti-inflammatory and anti-neogenesis effects of Dapagliflozin systemically and on the peritoneal membrane by observing serum Interleukin-6 (IL-6), serum Vascular Endothelial Growth Factor (VEGF), and peritoneal VEGF.

## Methods

### Study design and patients

**Fig. 1:**
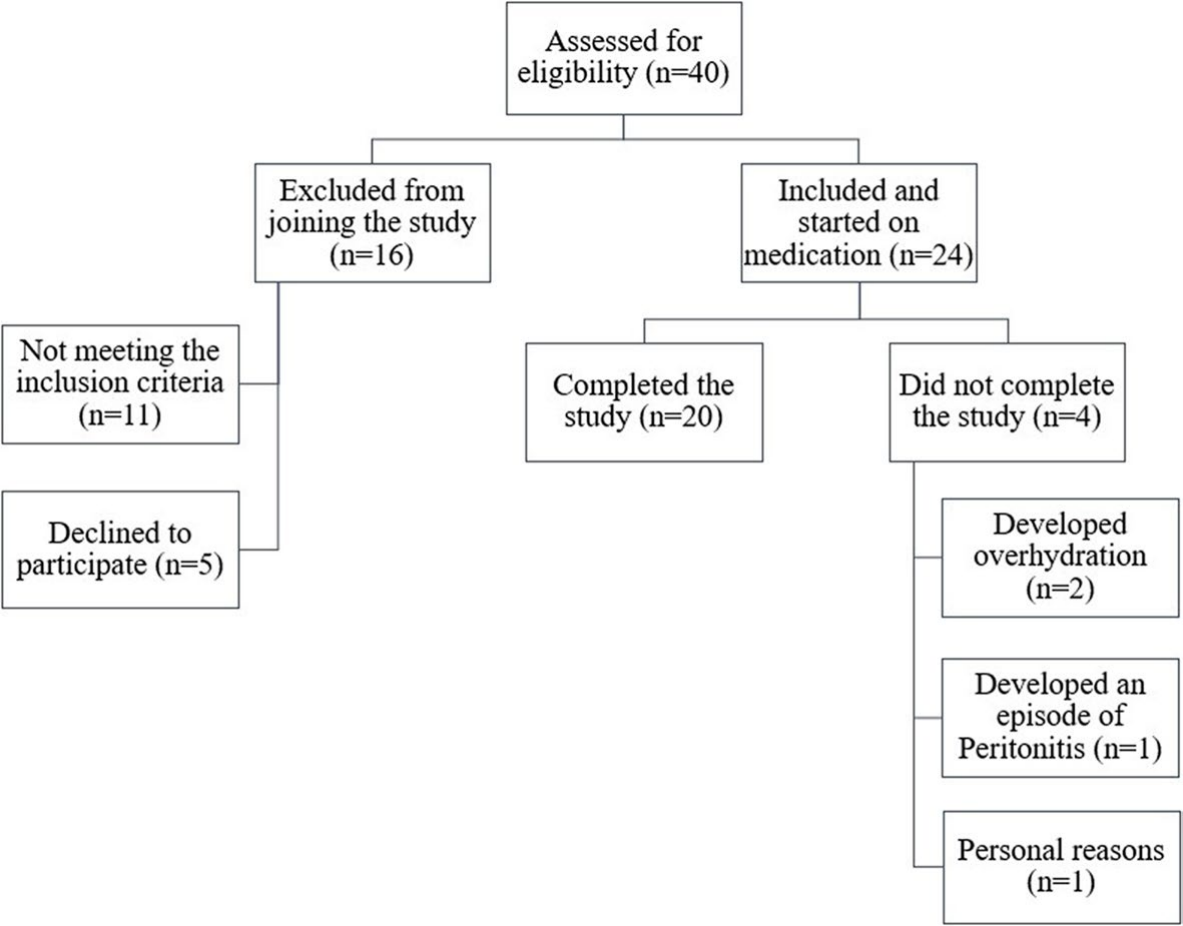
Sample selection and assessment flow chart – methodology

This pre-post pilot intervention study included 24 patients on CAPD (Fresenius Medical Care, stay•safe®), who were enrolled in a one-month retrospective self-controlled study, with 20 patients completing the study. The number of patients assessed initially to join the study was 40, with 16 patients excluded from joining due to either not meeting the inclusion criteria or refusing to participate (Fig. 1). Inclusion criteria included being over 18, having a PD vintage of at least six months, and using at least two exchanges with 2.35% dextrose over the previous three months before enrollment, having a high or high average peritoneal transport status based on a modified 4.5% Dextrose Peritoneal Equilibrium Test; with a D4/D0 less than 0.39. All participants had been on CAPD with four 2-L manual exchanges a day. In addition, patients who refused to participate in the study had a history of type I DM, peritonitis, urinary tract infection, any other infections within three months of the enrollment, history of recurrent hypoglycemia, liver disease, allergic reactions to SGLT-2 inhibitors, or any malignancy were excluded.

### Drug administration, testing, and follow-up

The participants were given 10 mg of Dapagliflozin daily for 30 days. A modified Peritoneal Equilibration Test (modified PET) with a 2-L 4.25% Dextrose [9] was performed at baseline and the end of the study period. Sodium peritoneal fluid measurements were done by the indirect electrode method. Ultrafiltration, weight, and blood pressure readings were recorded at the baseline modified PET and at the repeat PET one month after treatment. Plasma was collected at baseline and the end of the treatment period for IL-6 and VEGF. PD fluid was analyzed for VEGF at baseline and the end of the study. The IL-6 and VEGF concentrations were measured by Abcam’s ELISA kits in Cambridge, MA, USA (ab178013, ab100662), respectively. No changes were made to the PD prescription or to the participants' medications during the study period. All mandatory laboratory health and safety procedures have been complied with in the course of conducting the trial. Investigators were in contact with patients every week to evaluate any reported events and changes, such as hypoglycemic events, changes in blood pressure, or weight changes. Participants were also instructed to contact the PD unit for any concerns or any new changes during the study period.

All procedures performed in this study have been carried out following the Declaration of Helsinki and relevant national guidelines and regulations. The study protocol was approved by the Institutional Review Board committee of An Najah National University [Reference#: Med. April 2021/13] and registered under ClinicalTrials.gov [Trial#: NCT04923295]. Patients were informed of the purpose, objectives, and potential risks of the study before being asked to participate and sign an informed consent voluntarily. The confidentiality and privacy of the participants were protected. The information gathered was only accessible to members of the research team.

The primary outcomes of this study included: 1. changes in D4/D0 after one-month treatment with 10 mg of Dapagliflozin daily, 2. changes in Peritoneal Solute Transfer Rates (PSTR) measured by D/P, 3. Changes in sodium dip, 3. Changes in the net ultrafiltration volume, 4. Changes in weight, 5. Effect of Dapagliflozin on the inflammatory marker (IL-6) and vascular growth marker (VEGF). The Secondary outcomes were changes in blood pressure, development of infections, hypoglycemic episodes, and alterations in bicarbonate levels and other biomarkers.

### Data collection

Before Dapagliflozin administration, baseline demographic and clinical parameters, including age, gender, PD vintage, body mass index (BMI), DM status, HD vintage, first modality of dialysis, history of HD venous access failure, and laboratory data, were collected and measured. The dialysate to plasma creatinine (D/P) was calculated as the ratio of dialysate creatinine concentration after a four-hour dwell with 4.25% dextrose PD solution (Modified PET) to the serum creatinine concentration. The dialysate glucose at 4 h to dialysate glucose at 0 h (D4/D0) was also calculated with a 4.25 dextrose PD solution. Peritoneal ultrafiltration (PUF) was calculated as the difference between the PD solution installation volume and the volume of the PD solution drain after a 4-h dwell time. The Sodium Dip was calculated as the absolute difference between dialysate sodium before and one hour following dwell time. Blood pressure (BP), heart rates, weight, and inflammatory markers, such as IL-6, serum VEGF, and peritoneal VEGF, were collected at the start and end of the study.

### Data analysis

SPSS statistical software was used for data entry and analysis. Descriptive statistics were conducted with frequency and percentages for categorical variables and median (range) for continuous variables. Changes in D/P, D4/D0, PUF, and Na Dip were analyzed individually and as a group under a non-related parametric samples test. In addition, IL-6, peritoneal VEGF changes, and serum VEGF were also analyzed as a group under the non-related parametric samples test. The significance level was set at a *p*-value ≤ 0.05.

## Results

### Baseline characteristics

The demographic characteristics of the study participants show a median age of 49.5, a gender female: male ratio of 13:7, a 40% incidence of diabetics, a median BMI of 26.9 kg/m2, and a median PD vintage of 11.5 months. The majority (85%) had no urine output reported. Only three patients had PD as their first renal replacement modality, while 17 patients had been on HD before starting PD (Table 1).

**Table 1:** Subjects’ background and clinical characteristics

	**Frequency (%)**	**Median [Range]**
***Gender***		
Male	7 (35%)	
Female	13 (65%)	
***Age (years)***		49.5 [22–77]
***BMI (should use weight not BMI)***		26.9 [19.6–40.6]
***Diabetes Mellitus***	8 (40%)	
***Hypertension***	14 (70%)	
***HD access failure prior to PD initiation***	16 (80%)	
***First dialysis modality***		
Hemodialysis	17 (85%)	
Peritoneal Dialysis	3 (15%)	
***HD duration prior to PD (months)***		120 [18–240]
***PD duration (months)***		11.5 [5–56]
***Residual urine***	3 (15%)	
***History of peritonitis***	6 (30%)	

### PET results and sodium dip prior to and following Dapagliflozin treatment

**Fig. 2:**
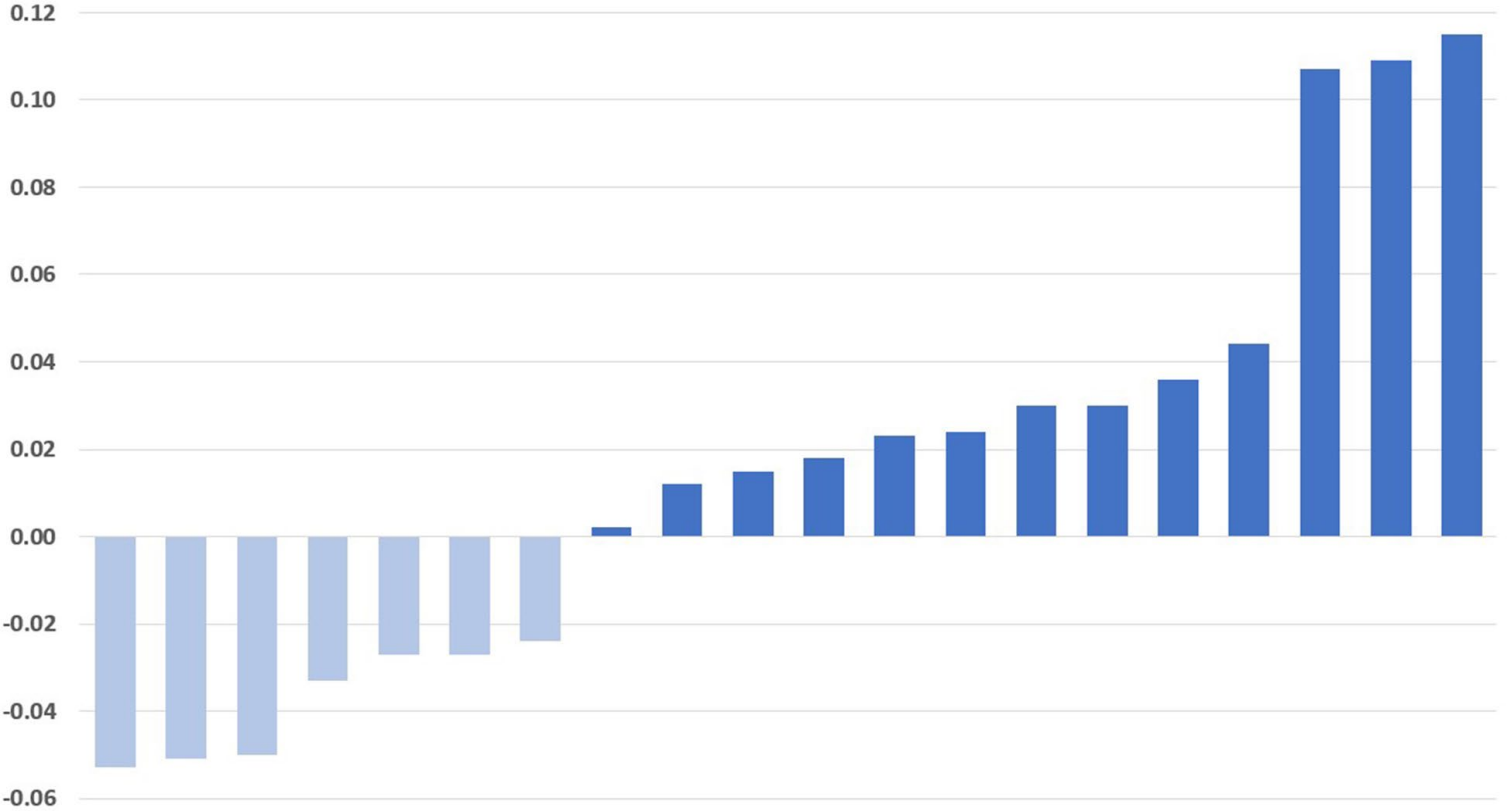
Changes in glucose D4/D0 after 30 days of treatment with Dapagliflozin in CAPD patients. Modified PET with 4.25% Dextrose was performed before and 30 days after the daily dose of 10 mg Dapagliflozin. After treatment with Dapagliflozin, 13 patients had an increase of D4/D0 (dark blue) while 7 patients had a decrease (light blue). Change in of D4/D0 = [Post-treatment]—[Pre-treatment]. CAPD: Continuous Ambulatory Peritoneal Dialysis

**Fig. 3:**
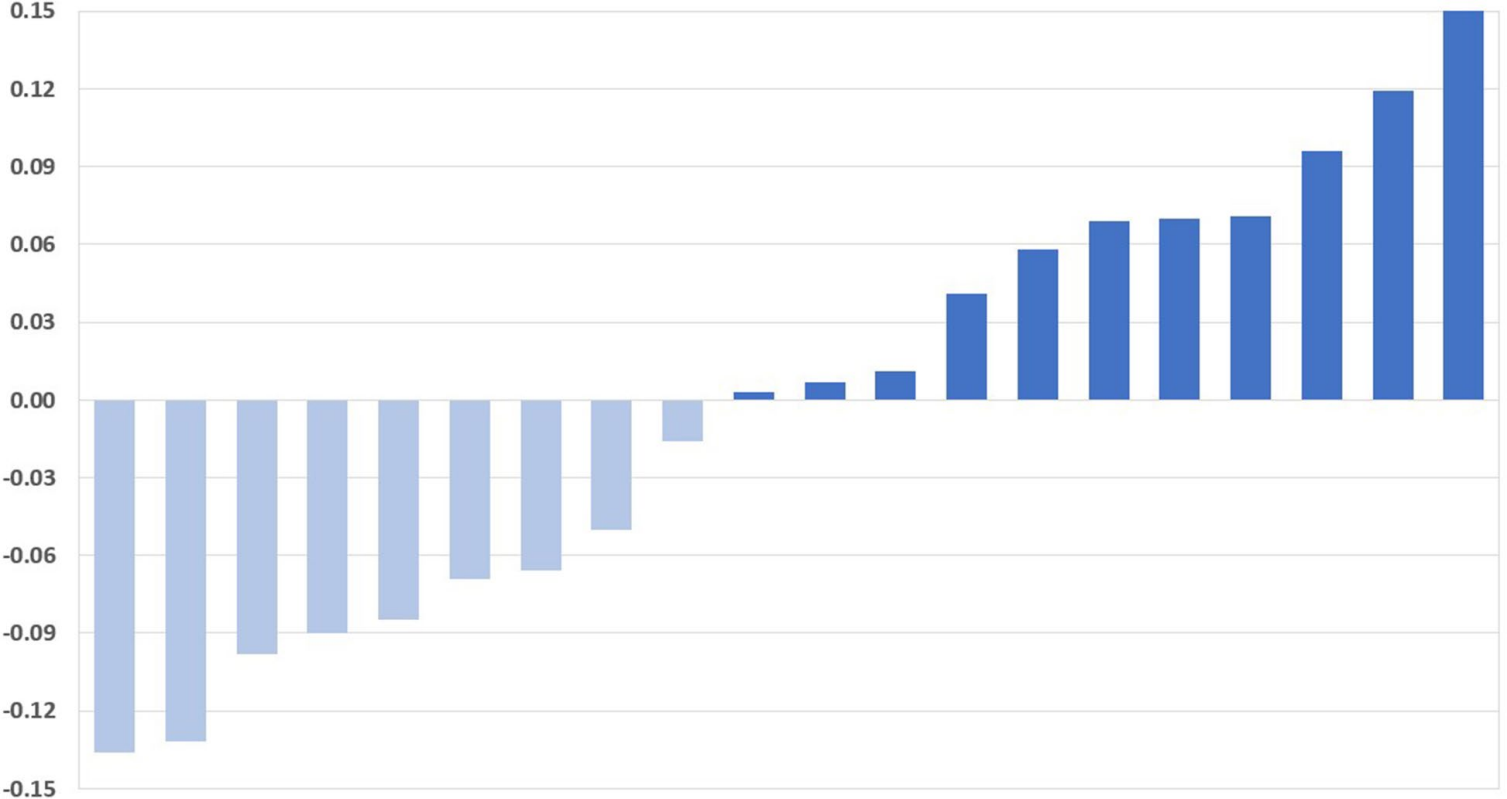
Changes of Creatinine D/P after 30 days of treatment with Dapagliflozin in CAPD patients. Modified PET with 4.25% Dextrose was performed before and 30 days after the daily dose of 10 mg Dapagliflozin. After being treated with Dapagliflozin, 9 patients had a reduction of D/P (light blue) and 11 patients had an increase in D/P after treatment (dark blue). Change in D/P = [Post-treatment]—[Pre-treatment]. CAPD: Continuous Ambulatory Peritoneal Dialysis

**Fig. 4:**
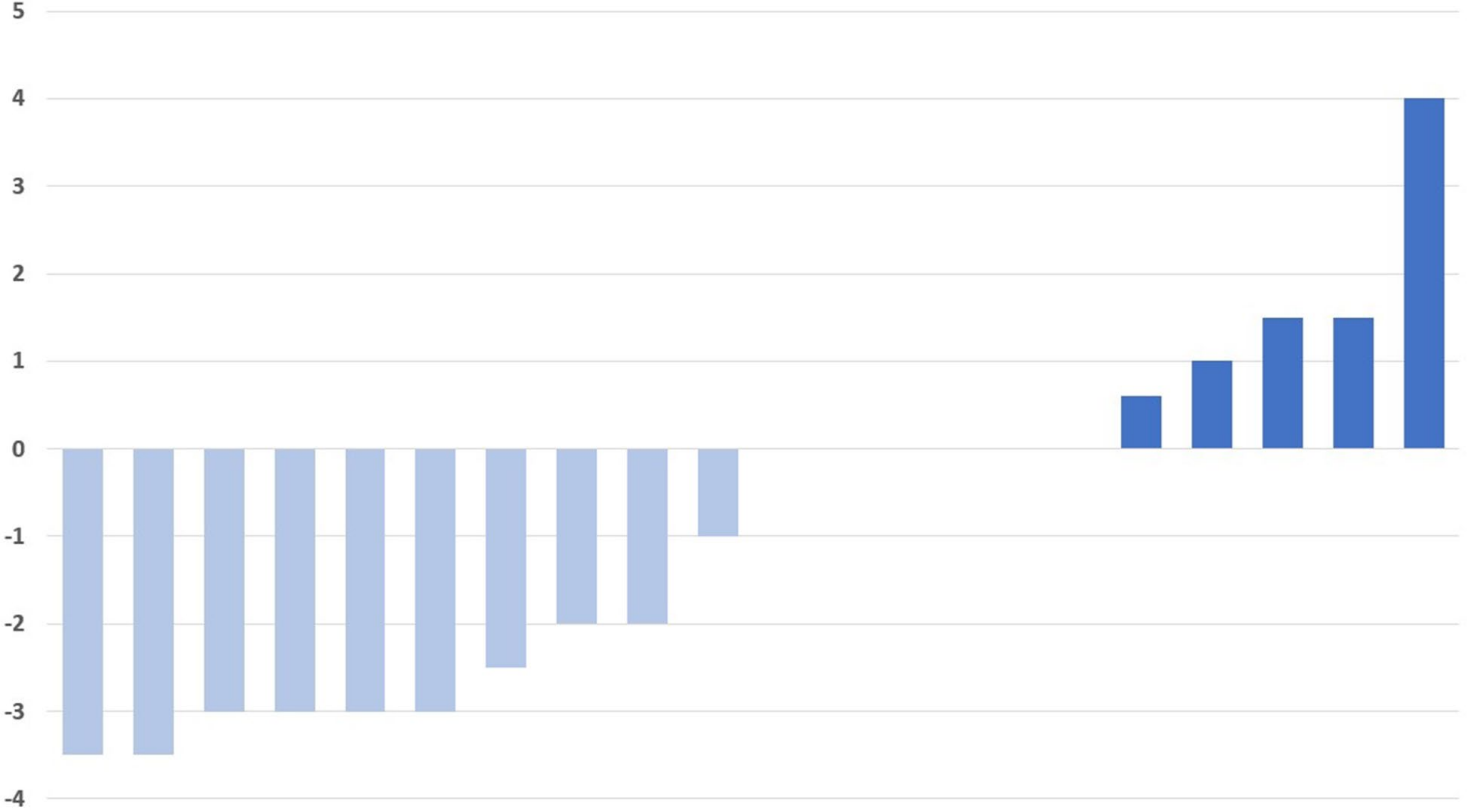
Changes in patients’ weight (Kg) after 30 days of treatment with 10 mg Dapagliflozin daily. After being treated with Dapagliflozin, 5 patients had an increase in their weight (dark blue), 10 patients had a reduction in their weight (light blue), and 5 patients had no weight change following the treatment. Change in Weight = [Post-treatment]—[Pre-treatment]. CAPD: Continuous Ambulatory Peritoneal Dialysis

To evaluate the effects of Dapagliflozin on the peritoneal membrane, we analyzed the changes of D4/D0, D/P, total ultrafiltration, and patients’ weight changes. After one month of Dapagliflozin treatment, 13 patients had an increase in median D4/D0 from 0.26 to 0.31, while seven patients had a decrease in median D4/D0 from 0.28 to 0.23 (Fig. 2). Moreover, nine patients showed a decrease in median D/P from 0.88 to 0.81, while 11 patients showed an increase in median D/P from 0.70 to 0.76 (Table 2) (Fig. 3). Furthermore, five patients showed an increase in weight with a median weight gain of 1.5 kg, and ten patients showed a decrease in weight with a median weight loss of -3 kg (Table 2) (Fig. 4).

**Table 2:** Individual analysis of patients with an increased D4/D0 after Dapagliflozin administration

Patient #	Age	Gender	BMI	Diabetic Status	History of Peritonitis	D4/D0		D/P		Na Dip		Weight	
						**Before**	**After**	**Before**	**After**	**Before**	**After**	**Before**	**After**
**2**	65	F	40.6	+	-	0.31	0.34	0.61	0.63	15	10	104.4	105
**3**	55	F	36.6	+	+	0.38	0.4	0.67	0.57	16	16	115	112
**4**	47	M	31.8	+	-	0.22	0.25	0.89	0.82	6	6	80	81.5
**6**	52	M	29.4	+	+	0.21	0.23	0.88	0.83	12	10	92.5	90
**8**	77	F	22.3	+	-	0.26	0.31	0.8	0.87	6	6	57	57
**12**	59	M	25.6	+	-	0.17	0.27	0.9	0.81	4	7	78	82
**1**	47	F	24.5	-	+	0.19	0.41	0.81	0.67	7	6	69.5	66
**7**	36	F	22.1	-	+	0.28	0.32	0.7	0.69	11	11	47.5	44
**11**	45	F	22.7	-	-	0.26	0.37	0.67	0.54	10	12	59	58
**13**	50	F	24.3	-	-	0.32	0.33	0.64	0.65	8	11	73	74.5
**14**	44	M	28.4	-	-	0.3	0.32	0.7	0.71	6	7	90	87
**15**	37	M	19.6	-	+	0.23	0.25	0.7	0.77	14	11	58	58
**17**	22	F	36.6	-	-	0.26	0.27	0.89	0.81	4	10	92	89

A collective set analysis was performed using a non-parametric related samples T-test with a calculated median for the variables before and after the experimental treatment. The variables assessed included the PET results, sodium dip, weight change, serum IL-6, serum VEGF, and fluid VEGF. Urea D/P at 4 h stood at a median of 0.95 prior to treatment and 0.95 following treatment, while pre-treatment creatinine D/P at 4 h stood at a median of 0.73 with a post-treatment median standing at 0.77. Additionally, glucose D4/D0 had a pre-treatment median of 0.263 and a post-treatment median of 0.29, and considering the importance of this variable in determining treatment efficacy, an individual analysis showcasing the age, gender, weight, BMI, diabetic status, history of peritonitis, PET results, and sodium dip of the patients was performed for those with an increased glucose D4/D0 (Table 3).

**Table 3:** Laboratory and clinical markers—results of interest

	**Pre-treatment** ***Regular Dialysis***	**Post-treatment** ***SGLT-2 Dialysis***	*P*-value*
	***Median [Range]***		
***D/P***	0.732 [0.596–0.921]	0.767 [0.537–0.913]	0.940
***D4/D0***	0.263 [0.165–0.384]	0.290 [0.143–0.396]	0.433
***Sodium Dip***	6.5 [1.0–16.0]	7.0 [3.0–16.0]	0.587
***Ultrafiltration***	715 [-500–1225]	700 [320–1160]	0.322
***Weight***	77 [47.5–115]	77.5 [44–112]	0.087
***Serum IL-6***	5 [0–22.2]	0 [0–18]	0.084
***Fluid VEGF***	9 [0–72.5]	2.5 [0–127]	0.349
***Serum VEGF***	185 [60–475]	210 [63–478]	0.394

^*^Non-Parametric Related Samples Test

Analysis of post-treatment sodium dip and ultrafiltration has shown a median of 7.0 and post-treatment sodium dip of 6.5. We have also assessed various inflammatory markers and have found that serum IL-6, fluid VEGF, and serum VEGF held a pre-treatment median of 5, 9, and 185, respectively, and a post-treatment median of 0, 2.5, and 210, respectively. The P-value under the non-parametric related samples T-test for the variables indicating the results of interest has shown no statistical significance (Table 4).

**Table 4:** Laboratory and clinical markers—safety indicators

	**Pre-treatment** ***Regular Dialysis***	**Post-treatment** ***SGLT-2 Dialysis***	*P*-value*
	*Median [Range] (Missing)*		
***Systolic Blood Pressure***	125 [70–179]	112 [80–193]	0.904
***Diastolic Blood Pressure***	77.5 [40–90]	72 [52–95]	0.481
***Serum Creatinine***	10.55 [6.40–17.39]	10.89 [6.06- 17.98]	0.135
***Serum Bicarbonate***	21.70 [18.40–25.30]	21.55 [16.30–26.10]	0.936
***Serum Uric Acid***	6.40 [4.10–9.30]	6.70 [3.90–8.50]	0.190
***Serum Blood Urea Nitrogen***	47.70 [30.10–99.90]	46.60 [33.40–100]	0.211
***Serum Albumin***	3.57 [2.25–4.89]	3.43 [2.62–4.99]	0.390

^*^Non-Parametric Related Samples Test

### Safety indicators – laboratory and clinical markers

Various clinical markers and laboratory tests were performed throughout the treatment duration to monitor the safety of our patients without any reported episodes of hypoglycemia, dysuria, or changed urinary frequency. One patient developed an episode of peritonitis 20 days after the start of the treatment, and two patients experienced overhydration a few days after the treatment. All three patients were excluded from the study. Serum bicarbonate has not significantly changed throughout the study.

## Discussion

Dapagliflozin’s primary mechanism of action is the inhibition of the sodium-glucose co-transporter 2 with its use in advanced Chronic Kidney Disease (CKD) and ESRD recently demonstrated in peritoneal dialysis patients with type 2 Diabetes More recently a case study involving incident peritoneal dialysis patients has revealed an improvement of Ultrafiltration with the use of Dapagliflozin [10]. Evidence has emerged supporting the theory of SGLT-2 expression in the human peritoneum and the upregulation of SGLT-2 receptors in patients with encapsulating peritoneal sclerosis [11]. Recent investigations have also revealed an improvement in diabetic control and an increase in ultrafiltration of PD patients after a six-month follow-up on Dapagliflozin, and a reduction in inflammatory markers, both in serum and PD effluent [12]. Mice studies have been contradictory, with one study demonstrating that inhibition of SLGT-2 by Dapagliflozin reduced glucose reabsorption by the peritoneum, while another study using empagliflozin did not result in a significant change in glucose, sodium, or water transport [8].

Ultrafiltration insufficiency limits the utilization of peritoneal dialysis for patients with no other options for renal replacement therapy. In addition, UF insufficiency can result in significant mortality; hence, we wanted to study the effect of Dapagliflozin on the peritoneal membrane function during a one-month treatment with the SGLT-2 Inhibitor Dapagliflozin in patients with a mean D/P of 0.73 who could be classified as having a fast PSTR. The study group also had a median D4/D0 of 0.26, which could also be classified as having a high average transport status. With our study showing an increase in D4/D0 from 0.26 to 0.29, which was not statistical significance likely due to the small sample size.

Regarding the change in glucose absorption, there was no statistical significant change despite a reduction in glucose absorption noted in some patients. One possible explanation is the genetic variability of the Aquaporin-1 promoter receptors among patients [13]. The reduction of sodium dip has been identified as an independent predictor of developing ultrafiltration insufficiency [14] and, thus, worse outcomes in PD. LA Millia et al. have shown that increasing the sodium dip by one mmol/L reduces the risk of developing UF insufficiency [14].The results of our study showed a small increase in sodium dip after Dapagliflozin treatment which was not statistically significant.

Peritoneal membrane longevity in PD is limited by glucotoxicity, which is hypothesized to be secondary to the degradation of glucose that changes the intracellular NADH/NAD + ratio resulting in pseudohypoxia, and, thus, inducing peritoneal fibrosis via the stimulation of the Hypoxia-Inducible Factor-1 gene among other genes, such as Transforming Growth Factor Beta, Connective Tissue Growth Factor, Plasminogen Activator Inhibitor-1, and VEGF [15]. One strategy to diminish this pseudohypoxia is to reduce peritoneal membrane exposure to a high dialysate glucose load. We hypothesized that adding Dapagliflozin would result in a reduced peritoneal intracellular degradation of glucose and, thus, a lower NADH/NAD + ratio. This was evident as there was a reduction in the peritoneal effluent VEGF before and after treatment with Dapagliflozin. VEGF has been shown to be associated with transport function status, so a reduction in intraperitoneal VEGF may result in less neogenesis [16]. 

SGLT-2 inhibitors have been previously evaluated for their possible anti-inflammatory effects, as Aso et al. has reported a significant reduction of IL-6 in their study [17]. Furthermore, a systemic review of 30 studies on using SGLT-2 inhibitors in rodent models also showed a reduction of IL-6 [18]. Therefore, our study also supports the possible anti-inflammatory effects of Dapagliflozin on systemic IL-6 levels.

Regarding the safety measures, our study administering SGLT-2 inhibitors to end-stage renal disease patients has been designed with appropriate measures to monitor and minimize any potential health risks to the patients that participated in the study. In addition to reviewing all safety details from all clinical studies that used Dapagliflozin, our patients had no residual renal function and no or minimal urine output with continuous daily clearance by peritoneal dialysis, and, therefore, the possibility of hypoglycemia, metabolic acidosis, and urinary tract infections was minuscule. During the one month of Dapagliflozin administration, there were no reported episodes of hypoglycemia or hypotension. No worsening acidosis was noted, and no significant changes were in any laboratory parameters (Table 4). However, two patients reported overhydration after a few days of use, and one developed an episode of peritonitis. The medication was discontinued for all three patients, and they were dropped from the study.

Regarding the limitations of our study, the sample size of participating patients consisting of 20 patients can be regarded as a small sample. Some additional limitations include the short treatment duration of one month, which limits the study's power, and the absence of a control group. Therefore, we recommend conducting further large controlled trials to evaluate the possible benefits of SGLT-2 inhibitors on peritoneal dialysis patients.

## Conclusion

This pilot study did not show a statistically significant increase in D4/D0 and thus no reduction in glucose absorption across the peritoneal membrane. Furthermore, Dapagliflozin was associated with a small non statistical increased sodium dip. It is also apparent that Dapagliflozin administration in PD patients may reduce systemic IL-6 and may result in a lower production of peritoneal membrane VEGF, resulting in reduced angiogenesis and possible protection of the peritoneal membrane. Therefore, its long-term effects on the longevity of the peritoneal membrane warrant further investigations.

## Data Availability

The datasets used and analyzed during the current study are available from the corresponding author on reasonable request.

## Abbreviations

CAP: Continuous Ambulatory Peritoneal Dialysis
ESRD: End-Stage Renal Disease
HPMC: Human Peritoneal Mesothelial Cells
IL-6: Interleukin-6
PD: Peritoneal dialysis
PSTR: Peritoneal Solute Transfer Rates
SGLT-2: Sodium-Glucose Transport Protein 2
VEGF: Vascular Endothelial Growth Factor
UF: Ultrafiltration Failure
